# Cannabidiol in Neurological and Neoplastic Diseases: Latest Developments on the Molecular Mechanism of Action

**DOI:** 10.3390/ijms22094294

**Published:** 2021-04-21

**Authors:** Marcin Ożarowski, Tomasz M. Karpiński, Aleksandra Zielińska, Eliana B. Souto, Karolina Wielgus

**Affiliations:** 1 Department of Biotechnology, Institute of Natural Fibres and Medicinal Plants—State Research Institute, Wojska Polskiego 71B, 60-630 Poznań, Poland; marcin.ozarowski@iwnirz.pl (M.O.); karolina.wielgus@iwnirz.pl (K.W.); 2 Chair and Department of Medical Microbiology, Poznań University of Medical Sciences, Wieniawskiego 3, 61-712 Poznań, Poland; 3 Institute of Human Genetics, Polish Academy of Sciences, Strzeszyńska 32, 60-479 Poznań, Poland; aleksandra.zielinska@igcz.poznan.pl; 4 Department of Pharmaceutical Technology, Faculty of Pharmacy, University of Coimbra, Pólo das Ciências da Saúde, Azinhaga de Santa Comba, 3000-548 Coimbra, Portugal; ebsouto@ff.uc.pt; 5 CEB—Center of Biological Engineering, University of Minho, Campus de Gualtar, 4710-057 Braga, Portugal

**Keywords:** *Cannabis sativa*, cannabidiol, CBD, mechanism of action, Alzheimer’s disease, epilepsy, multiple sclerosis, cancer, pharmacological activity

## Abstract

As the major nonpsychotropic constituent of *Cannabis sativa*, cannabidiol (CBD) is regarded as one of the most promising therapeutic agents due to its proven effectiveness in clinical trials for many human diseases. Due to the urgent need for more efficient pharmacological treatments for several chronic diseases, in this review, we discuss the potential beneficial effects of CBD for Alzheimer’s disease, epilepsy, multiple sclerosis, and neurological cancers. Due to its wide range of pharmacological activities (e.g., antioxidant, anti-inflammatory, and neuroprotective properties), CBD is considered a multimodal drug for the treatment of a range of neurodegenerative disorders, and various cancer types, including neoplasms of the neural system. The different mechanisms of action of CBD are here disclosed, together with recent progress in the use of this cannabis-derived constituent as a new therapeutic approach.

## 1. Introduction

It has been long recognized that *Cannabis sativa* L. has a great potential for medical application for different human diseases [1,2]. Its first medical use was marked in Central Asia, including China and India, where *C. sativa* was used to treat several conditions nearly five millennia ago [3]. Nowadays, the therapeutic potential of phytocannabinoids and unique extracts from *C. sativa* have been the focus of interest of multiple research groups worldwide. The latest scientific reports have opened the potential therapeutic use of the main *C. sativa* compounds, such as delta-9-tetrahydrocannabinol (Δ^9^-THC) and cannabidiol (CBD). The American Food and Drug Agency (FDA) approved dronabinol and nabilone to treat chemotherapy side-effects (nausea and vomiting) and for appetite stimulation in wasting diseases. In 2018, CBD was approved for the treatment of two types of pediatric epilepsy, namely, Dravet syndrome and Lennox–Gastaut syndrome.

**Figure 1:**
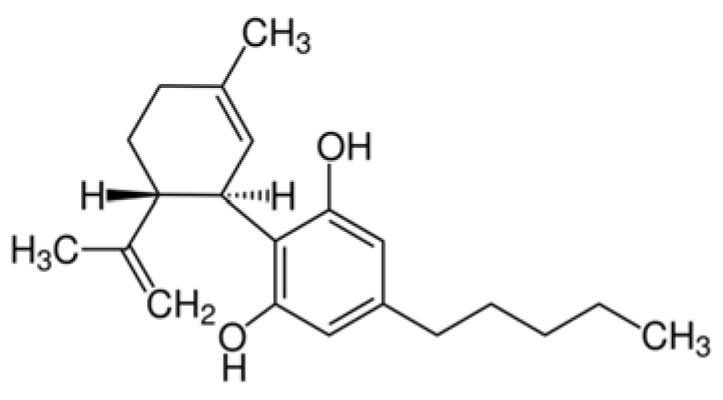
The structural formula of cannabidiol (CBD, C_21_H_30_O_2_).

Cannabidiol is one of the major nonpsychotropic constituents of *C. sativa* and it is considered a promising therapeutic agent due to its already proven effectiveness against neurological diseases [1,2,4,5]. CBD belongs to the group of terpenophenols. This bioactive compound consists of 21 carbon atoms and, according to International Union of Pure and Applied Chemistry (IUPAC) terminology, is described as 2-[(1R,6R)-3-methyl-6-prop-1-en-2-ylcyclohex-2-en-1-yl]-5-pentylbenzene-1,3-diol [6]. The structural formula for CBD is shown in Figure 1. The chemical activity of this *Cannabis sativa*-derived compound is based on the location of hydroxyl groups either in the phenolic ring (at the C-1′ and C-5′ positions) and the methyl group at the C-1 position of the cyclohexene ring and the pentyl chain at the C-3′ of the phenolic ring. On the other hand, the ring of CBD is inactive in the C-4 position [6]. Thanks to hydrogen bonds, CBD has an affinity to amino acids (including threonine, tyrosine, glutamic acid, glutamine). Besides its activity in the central nervous system (CNS), other properties (e.g., anti-inflammatory, antinausea, antitumor, anticonvulsant and anxiolytic), have also been reported [7].

In this review, the current state of the art knowledge is discussed with respect to the molecular mechanisms of action of CBD in Alzheimer’s disease (AD), epilepsy, multiple sclerosis, and the role of CBD in compromising the pathological process of these diseases. The impact of CBD on many types of cancer, including neoplasms of the neural system, is also addressed.

## 2. Materials and Methods

In this review, publications available in PubMed and Scopus databases and available through the Google search engine were taken into account. The following keywords and their combinations were used: “cannabidiol”, “CBD”, “Alzheimer’s disease”, “epilepsy”, “multiple sclerosis”, “cancer”, “neural system”. Additional searches included references from identified publications. In the screening process, articles published in predatory journals and research before 1980 were excluded.

## 3. Recent Developments on the Molecular Mechanism of Action of CBD for Alzheimer’s Disease

CBD is a potential bioactive for the treatment of neuroinflammatory-derived diseases, epilepsy, anxiety, and schizophrenia [8]. Due to its antioxidant, anti-inflammatory, and neuroprotective properties, cannabidiol was proposed as a promising innovative strategy for Alzheimer’s disease [9,10]. Moreover, CBD reduces the hyperphosphorylation of tau protein [9], inhibits acetylcholinesterase activity [11], and deposition and expression of beta-amyloid [10,12,13]. These pharmacological points of action are involved in the complex pathogenesis of Alzheimer’s disease. However, the molecular mechanisms of CBD in this field are still to be fully described. A study by Huges et al. [14] described that CBD (10 μM) rescued the deficit in long-term hippocampal potentiation in c57/black six mice induced by β amyloid (Ab 1–42) by a receptor-independent mechanism, such as 5HT1A, adenosine A2A, and the receptor CB1. CBD pharmacological activities were also reported to be related to diverse non-CB1/non-CB2 mechanisms [15,16].

**Figure 2:**
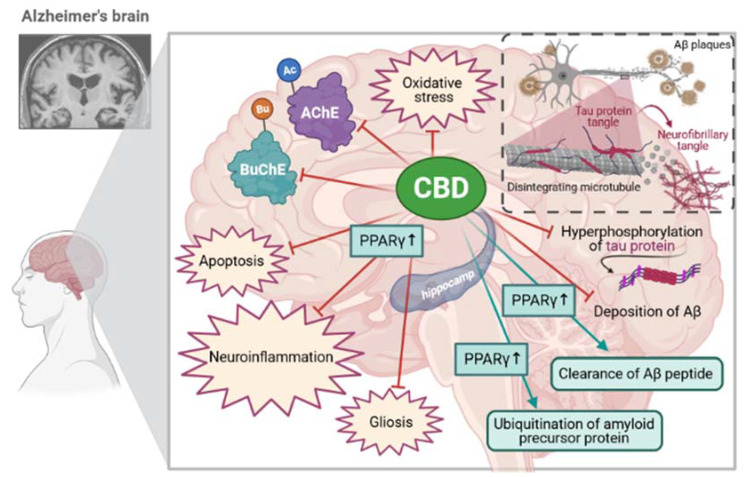
The role of CBD in the pathogenic mechanism of the Alzheimer’s disease [own drawing]. Legends: CBD: Cannabidiol; AChE: Acetylcholinesterase; BuChE: Butyrylcholinesterase; Aβ: beta-amyloid; PPAR-γ: peroxisome proliferator-activated receptor-gamma.

The molecular mechanism of action of CBD is nowadays attributed to its influence on the activity of peroxisome proliferator-activated receptor γ (PPAR-γ), which belongs to the nuclear hormone receptor superfamily, and its transcription factors are activated by ligand [17]. CBD can act as a PPAR-γ agonist [18]. Studies carried out in animal models of Alzheimer’s disease showed that PPAR-γ agonist caused, i.e., the reduction of amyloid plaque and inflammation. Furthermore, the agonist of this receptor improved cognitive and memory function in patients with mild to moderate Alzheimer’s disease [19]. For this reason, CBD can be an interesting therapeutic intervention [19]. Several studies demonstrated that CBD exerts antiapoptotic activity, increases the ubiquitination of amyloid precursor protein [12], enhances the clearance of Aβ peptide [20], showing anti-inflammatory and antigliosis properties [21], depending on the selective activation of PPAR-γ (Figure 2). For this reason, CBD can be an interesting therapeutic intervention.

### 3.1. The Effect of CBD on Acetylcholinesterase (AChE) and Cholinergic System in the Brain

Acetylcholinesterase participates in the hydrolytic breakdown of acetylcholine in the CNS and is a target-site for cannabidiol and other bioactive chemical compounds of *Cannabis sativa* L. [22,23,24,25]. Recent studies have focused on explaining the basis of the interaction between CBD and acetylcholinesterase (AChE), a key enzyme in the pathogenesis of Alzheimer’s disease [11,26]. However, many years ago, it was discovered that cannabinoids influence the limbic system by the modulation of cholinergic neuron activity in the hippocampal area [27].

#### 3.1.1. Mechanism of Action of CBD

Recently, Furqan et al. [11] used the blood of humans addicted to *Cannabis sativa* to estimate the activity of AChE by performing an in silico study to assess the binding affinities with AChE. Analysis of the molecular docking of CBD to the binding sites of AChE showed that this cannabinoid inhibited AChE activity similarly to eight other cannabinoids. However, cannabidiol possessed the strongest effect attributed to its lipophilic properties. This in silico study confirms that CBD and other cannabinoids have promising therapeutic applications as inhibitors of AChE [11]. Moreover, another in silico study showed that all tested cannabinoids possessed the ability to inhibit both AChE and butyrylcholinesterase [25], which is a second enzyme involved in the pathogenesis of Alzheimer’s disease [28].

#### 3.1.2. Pharmacological Effects of CBD in CNS

Murillo-Rodríguez et al. [26] revealed that CBD given to rats in systemic injections (5, 10 or 30 mg/kg, i.p.) caused a dose-dependent effect, increasing the level of acetylcholine in the basal forebrain. A statistical significance effect was observed 6 h after injection of CBD at all doses; however, the highest dose (30 mg/kg) also showed the higher concentration of this neurotransmitter. Naranjo et al. [29] observed the influence of CBD on the muscarinic neurotransmission in particular sites of the rat brain involved in memory processes, in the prefrontal cortex and hippocampus, in which CBD (10 mg/kg for three weeks) caused a normalizing effect in the expression of the choline acetyltransferase and binding density of muscarinic M1/M4 receptor, which may have positive outputs in case of memory deficits. It has already been observed that various cannabinoids can increase acetylcholine and decrease its turnover in the cortex and hippocampus of the mouse brain [30].

### 3.2. Antiapoptotic Effects of CBD during Cognitive Decline

Da Silva et al. [31] conducted a study about the effect of CBD (10 mg/kg by intraperitoneal injection for 14 days) on the toxic effects of iron carbonyl causing severe memory deficits in rats referring to Alzheimer’s, Parkinson’s, and Huntington’s disease.

Molecular analyses of brains showed decreasing effects of CBD on the elevated level of caspase-9, caspase-3, and cleaved poly (ADP-ribose) polymerase-1 (PARP) caused by iron carbonyl and CBD completely reversed iron-induced effects on apoptotic protease activating factor 1 (APAF1) [31]. These mechanisms of action may suggest that CBD has antiapoptotic effects during neurodegeneration. Other authors reported that CBD (10^−7^ M) diminished apoptotic events in SHSY5YAPP+ cells through interaction with peroxisome proliferator-activated receptor γ (PPAR-γ) [12].

### 3.3. The Effect of CBD on the Beta-Amyloid Synthesis and Beta-Amyloid-Induced Toxicity

The accumulation of extracellular aggregates of amyloid-β (Aβ) peptides in senile plaques, found in various parts of the brain, is one of the key factors of pathogenesis and progression of Alzheimer’s disease [32,33] and other diseases, such as Down’s syndrome, dementia with Lewy bodies, corticobasal degeneration, chronic traumatic encephalopathy [34]. It should be emphasized here that an increasing number of studies are focused on the molecular interaction of CBD with the amyloid pathway *in vitro* and *in vivo* [12,13,14,35,36,37,38,39,40].

Scuderi et al. [12] tested various concentrations of CBD (10^−9^–10^−7^ M) in neuroblastoma cells SHSY5YAPP+ with an expression of full-length APP and, it was observed that CBD decreased fragments (C83 and C99) of amyloid precursor protein in a dose-dependent fashion and reduced the expression of Aβ peptide. Moreover, CBD can induce the ubiquitination of the amyloid precursor protein. These effects were dependent on the selective activation of peroxisome proliferator-activated receptor γ (PPAR-γ) and this molecular mechanism may have a crucial role during the amyloidogenic pathway because activation of PPAR-γ is associated with enhancing the clearance of Aβ peptide [20]. Reducing the expression of the Aβ peptide by CBD may affect the protection of neurons against the toxic effects of this peptide positively [12]. However, Janefjord et al. [37] did not show biochemical and morphological effects of the incubation of SH-SY5Y cells with β amyloid (Aβ 1–42) and the CBD (10 µM). Hao et al. [13] also observed that CBD was given to APP/PS1 mice at a dose of 5 mg/kg/d, i.p. during 30 days, decreased the level of the Aβ plaques in the hippocampus. On the other hand, Janefjord et al. [37] revealed that CBD prevented cell toxicity induced by Aβ peptide, similar to other authors.

### 3.4. The Effect of CBD on Hyperphosphorylated Forms of Tau Protein

Intracellular neurofibrillary tangles composed of aggregated protein tau are the second pathological hallmark of Alzheimer’s disease [35]. A few studies showed that hyperphosphorylated tau protein can be a promising target for the potential therapeutic strategy of CBD [13,21,36,39,40]. Previously, Esposito et al. [39] revealed that CBD (10^−7^–10^−5^ M) in PC12 cells incubated with Aβ inhibited the expression of the tau protein and the process of its hyperphosphorylation in a concentration-dependent fashion [39]. According to the authors, the observed effect is combined with a reduction in phosphorylated glycogen synthase kinase 3-β (p-GSK3-β), which further leads to the rescue of the Wnt/βcatenin pathway. It should be noted that p-GSK3-β (tau protein kinase) is responsible for the hyperphosphorylation of the tau protein and the formation of neurofibrillary tangles; thus, this molecular mechanism action may be essential in this target.

### 3.5. The Effect of CBD on Neuroinflammatory Processes

#### 3.5.1. Pharmacological Effects of CBD in CNS

Esposito et al. [38] reported that CBD (2.5 or 10 mg/kg, i.p.), given into the right dorsal hippocampus of mice for seven days with human Aβ peptide (1–42), decreased the expression of glial proinflammatory cytokine (IL-1β) and inducible nitric oxide synthase (iNOS). CBD also inhibited the mRNA expression of the glial fibrillary acidic protein (GFAP, a marker of activated astrocytes) in a dose-dependent manner. These studies thus confirm that CBD has neuroprotective, anti-inflammatory, and antigliosis properties.

#### 3.5.2. Mechanism of Action of CBD in the Hippocampus

Esposito et al. [21] reported that CBD (10 mg/kg) rescued CA1 pyramidal neurons in the hippocampus, induced neurogenesis in this part of the brain, and reduced reactive gliosis. The mechanism of action included the inhibition of nuclear factor-kappa β (NFkB), as revealed by the downregulation of p50 and p65; the selective activation of PPAR-γ; diminishing the expression of GFAP, and decreasing the release of NO, IL-1β, TNF-α. It was shown that CBD inhibited the proinflammatory activity induced by the Aβ peptide (1–42) by PPAR-γ [21]. Hao et al. [13] carried out an analysis of the transcriptome sequence (RNA-Seq) and differential expression analysis for the hippocampal tissues of brains of APP/PS1 mice after administration of CBD (5 mg/kg/d, i.p.) for 30 days. The results showed that CBD upregulated the immune response possibly by CB2 receptors occurring in glial cells. Moreover, CBD increased the autophagy in the hippocampus, which is a promising mechanism action of CBD because this process is involved in pathways for Aβ clearance. It was revealed that CBD increased the expression of autophagy-related proteins (Beclin1 and LC3). The effect of this mechanism of action of CBD was the decrease in the amyloid deposition in the hippocampus. Hao et al. [13] described 231 upregulated and 131 downregulated difference expression genes. These results require further analysis to draw more consistent conclusions.

#### 3.5.3. Mechanism of Action of CBD in Microglia Cells

CBD showed neuroprotective and anti-inflammatory properties by blockage of microglial activation [8,15,41,42]. CBD (20 mg/kg) was given to amyloid-injected mice for 3 weeks, decreased gene expression of IL-6 cytokine, decreased NO generation, and inhibited the increase in the intracellular calcium induced by ATP in cultured microglia [41]. Moreover, it was demonstrated that CBD (10^−6^ to 10^−4^ M) was able to inhibit NO production and expression of iNOS protein induced by Aβ peptide (1–42) in PS12 cells [40]. In the observed effect of CBD, the inhibition of phosphorylated form of p38 MAP kinase was involved in the activation of transcription factor -nuclear factor-kappa β (NFkB). Juknat et al. [15] reported that CBD diminished the LPS-dependent elevation of proinflammatory miRNAs associated with Toll-like receptor and NF-κB signaling, involving miR-146a, miR-155, and miR-34a.

The mechanism of action of CBD in Alzheimer’s disease is summarized below (Table 1).

## 4. Recent Developments on the Molecular Mechanism of Action of CBD for Epilepsy

As a highly heterogeneous neurological disorder, epilepsy belongs to neurological diseases described by spontaneous recurrent seizures [16,43]. These seizures have a form of brief or long episodes characterized either by early undetectable periods or by vigorous shaking [43]. However, they can be efficiently treated in most patients using one or more antiepileptic drugs [44]. Despite the advent of new medicines that have brought more advanced and effective treatments, these are still not able to remove the prevalence of drug-resistant epilepsy.

Nabbout and Thiele [45] have reported that CBD has been proven to have antiseizure activity in studies based on *in vitro* and in vivo models. Consequently, in 2013, GW Pharma has conducted the first phase of clinical trials using Epidiolex/Epidyolex (>99% CBD) known as a purified form of CBD. These trials have brought beneficial results of CBD efficiency and have shown high tolerability of this compound for the treatment of drug-resistant epilepsies. This, in turn, contributed to the approval as an effective drug of CBD by a US Food and Drug Administration (FDA) and the European Medicines Agency (EMA) in 2018 and 2019, respectively [46]. Both agencies have admitted that cannabis-derived compounds, such as CBD, are of great interest in the treatment of neurodegenerative diseases. Thereupon, cannabidiol has become the first FDA plant-derived compounds that can be successfully applied for the treatment of seizures in the various, rare epileptic disorders, namely: Lennox–Gastaut syndrome (LGS) or Dravet syndrome (DS) in two-year-old children (and older) with maximum doses of 10 mg/kg twice a day, and a maximum dosage of 50 mg/kg/day in adults [47]. Furthermore, the clinical trials with Epidiolex are regarded as the first open interventional exploratory study for LGS and DS. These studies have been carefully described by Nabbout and Thiele, who also mentioned the expanded access program in the USA including the treatment of CBD-resistant epilepsy in childhood and adults in the age range of 2–30 years [48]. Besides, Devinky et al. [49,50] has described clinical trials of CBD for the treatment of seizures in Dravet syndrome.

Meir and Perucca [51] have presented results that may confirm that CBD positively influences therapeutic effects in patients with epilepsy, either LGS or DS by reducing seizures. For this reason, several randomized placebo-controlled trials have been carried out as a therapy with CBD to control seizures in patients suffered from these epilepsies. According to the European Medicines Agency Public Assessment Report, all of the available evidence on the clinical consequences of the CBD influences were carefully observed and estimated by focusing on analyses of seizure outcomes in patients [51].

Lipnik-Štangelj and Razinger [52] have reviewed the actual legal framework in the European Union, which regulates cannabis-derived products for medical applications. The authors underline that although all of the requirements and marketing authorization procedures for medical products-based on plant-derived compounds for all EU were declared, there is no common regulatory framework for drugs-contained cannabinoids.

### 4.1. Mode of Action of CBD in Preclinical Development

The prevention of epilepsy development is also a great challenge for the current clinical practice, especially considering patients with a traumatic brain injury [44]. Preclinical development has proven that this bioactivity can be regarded as a potential antiepileptogenic agent. Its anticonvulsant effects tested in preclinical models highlighted the potential role of cannabis-derived constituents in treating epilepsy and its symptoms [53,54]. *In vitro* studies based on acute seizure models proved the anticonvulsant profile of CBD (in mice at 83.5 mg/kg up to 120 mg/kg, while in rats at 88.9 mg/kg). Preclinical tests using animal models of chronic epilepsy demonstrated the influence of CBD (up to 200 mg/kg) on the propagation of electrically kindled limbic seizures in rats [54].

### 4.2. Different Delivery Routes of CBD

The inhaled route remains the most widely used administration route for CBD. Plasma peak concentrations rapidly occurred after aerosolization or vaporization of CBD (200–300 mg/day, for 3–18 weeks), reaching about 31% of a final bioavailability [54]. Nevertheless, this administration route requires specialized equipment, therefore multiple approaches are focused on producing the most suitable and resistant form of drug delivery [54]. On the other hand, scientific trials have shown that CBD (up to 600 mg) delivered orally in oil-based capsules, resulted in about 6% bioavailability [55]. Due to its low water solubility, the absorption of CBD from the gastrointestinal system is compromised, resulting in very low bioavailability. Ethosomal delivery systems have been proposed to deliver CBD by transdermal route, favored by the compound’s high lipophilic character, thus preventing CBD accumulation in the skin [54,56].

### 4.3. The Impact of CBD on the Central Nervous System (CNS) in Preclinical and Clinical Trials

The focus on cannabis-derived products for the treatment of refractory epilepsy has significantly increased in recent years. As one of the major substitutes of *Cannabis sativa* L., CBD is recognized as a phytocannabinoid that can affect cannabinoid receptors, i.e., type 1 (CB1) and type 2 (CB2) that occur in the CNS and in the immune system [57]. Although almost a third of epilepsy patients are not effectively treated by the clinically available antiseizure drugs (ASDs), initial studies demonstrated that CBD-containing agents may be a useful treatment for pharmacoresistant epilepsy [58]. Perucca [53] reported that CBD has a well-defined anticonvulsant profile in animal models and it is mainly free from adverse psychoactive effects. The author also summarized a list of targets and actions reported for CBD. CBD may be an efficient agonist or antagonist of neurotransmitter transporters, of multiple noncannabinoid and transmembrane receptors, as well as of ion channels [59]. According to Klein et al. [58], CBD can show a positive impact on a broad spectrum of seizures. These results were obtained in the animal model. Ongoing clinical trials also indicate that therapeutic doses of purified CBD can be applied to patients suffering from epilepsy [5,45,60]. CBD is considered to have a great affinity for the treatment of multiple disorders, resulting in the modulation of neuronal excitability and synaptic transmission [59].

#### 4.3.1. Mechanisms of Action of CBD

Among the variety of mechanisms of action of cannabidiol, receptors including CB1 and G protein-coupled receptor 55 (GPR55), the transient receptor potential vanilloid 1 (TRPV1) with K^+^ channels and mitochondrial Na^+^/Ca^2+^ exchanger (NCX), are involved [14,17]. Due to the low affinity for endocannabinoid receptors by CBD, its mechanisms of action are mainly dependent on other molecular targets, such as TRPV1, to which the CBD shows a high affinity. TRPV1 is considered one of the most important ion channel targets [5]. The influence of CBD as an antagonist of CB1 allows the release of Gi/o protein-coupled with the reduction of TRPV1 activity. CBD can also interact with TRPV1, causing the inflow of Ca^2+^. Thereby a high calcium level can activate potassium channels, resulting in hyperpolarization of the presynaptic terminals. In turn, during the GPR55 activation [61], CBD as an antagonist may increase intracellular calcium via inositol 1,4,5-triphosphate (IP3) signaling. Furthermore, CBD can act on the NCX, reducing or elevating cytosolic calcium [59,62].

#### 4.3.2. Pharmacological Effects of CBD in CNS

**Figure 3:**
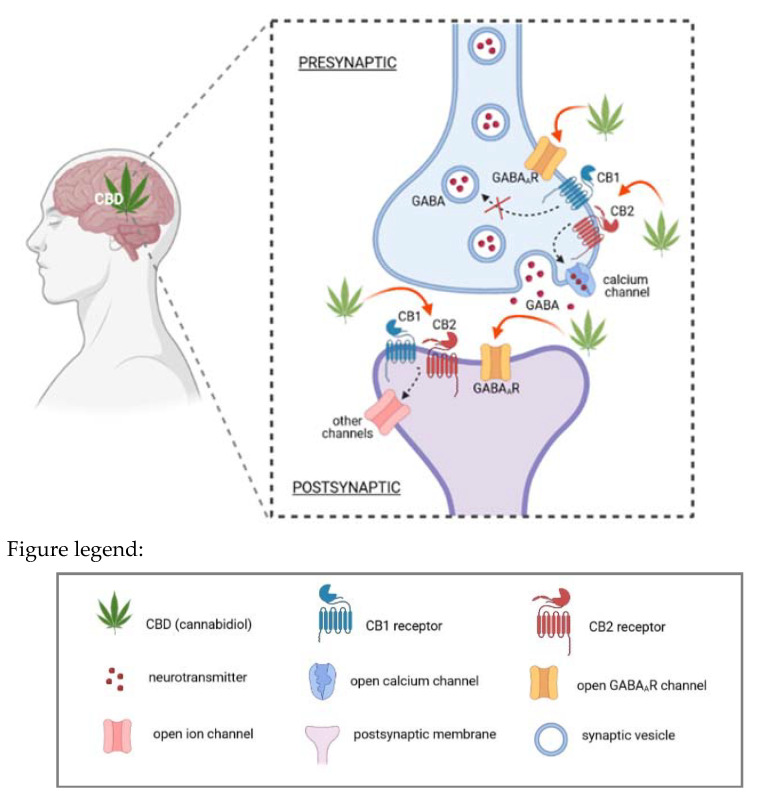
CBD mechanism of action in CNS own drawing, based on [64]. As an activator of cannabinoid receptors, CBD can modulate γ-aminobutyric acid type A receptor (GABA_A_R). As shown, CBD influences presynaptic cannabinoids receptors (CB1, CB2) and improves the postsynaptic activity of GABA. Cannabis-derived constituents (e.g., CBD, THC, CBDV) may occur both at the presynaptic and postsynaptic membranes.

The understanding of the pharmacological effects of CBD in CNS is still incomplete. Notwithstanding, recent scientific reports regarding the application of cannabis-derived constituents in different neurological disorders, show the important role of CBD in CNS, including the modulation of γ-aminobutyric acid type A receptors (GABA_A_Rs) both directly and through the activation of CB1 and CB2 [63,64]. As shown in Figure 3, CBD targets (including other cannabis-derived constituents, such as THC or CBDV) are placed both at the presynaptic and postsynaptic membranes. As described by Cifelli et al. [64], CBD is responsible for modulating GABA release. Thus, CBD has an impact on presynaptic cannabinoid receptors, and additionally, it can also improve the postsynaptic activity of GABA.

As multiple randomized controlled clinical trials confirmed that Dravet and Lennox–Gastaut syndromes respond to CBD, cannabinoid-based treatment is commonly recommended to minimize excitatory seizure [65,66]. It has been proven that CBD and cannabis-derived bioactives can reduce seizure attacks [65,66]. The mechanism of action is based on the presynaptic action on the equilibrative nucleoside transporter 1 (ENT1) adenosine pumps. Consequently, it may cause an increase in extracellular adenosine, while decreases in the GPR55 and TRPV1 receptors are also noticed [59,61].

### 4.4. Pharmaceutical Use of CBD

Rosenberg et al. [65] described cannabis-based antiepileptic therapies conducted in the framework of preclinical and clinical trials of cannabinoids in epilepsy. The obtained results suggested that CBD ensures a well-tolerated, promising therapeutic for the treatment of seizures, whereas a whole-plant cannabis can reduce seizures [65]. Furthermore, selected animal models have shown promising results concerning the antiseizure or antiepileptic effects of cannabinoids. Although the main clinical trials estimate the efficacy of cannabidiol in seizure control, a partial antiseizure effect of CBD has been shown. Thus, there is a constant need for follow-up placebo-controlled, blinded, randomized clinical trials assessing the role of CBD in seizure control [54,67].

## 5. Recent Developments on the Molecular Mechanism of Action of CBD for Multiple Sclerosis (MS)

As the most common chronic autoimmune disease of the CNS, multiple sclerosis (MS), also known as encephalomyelitis disseminate, is characterized by damaged insulating covers of the nerve cells in the brain and spinal cord [68,69]. The ability of signal transmissions in the nervous system has failed; therefore, multiple physical, mental, or even psychiatric problems may appear. Amongst the most typical symptoms, double vision, blindness in one eye, muscle weakness, and trouble with sensation or coordination, are mentioned [70,71]. According to scientific reports, cannabis-derived medicines can decrease MS symptoms, including spasticity, neuropathic pain, tremor, and disturbed bladder function [72,73]; however, no cannabinoids are indicated for the treatment of the pathophysiological basis of MS. There are currently no clinical trials to ensure fully efficient CBD-based therapies for spasticity and pain in MS and their successful endpoints. These results are still limited, despite the evidence in mouse studies of encephalomyelitis (EAE), which have shown CBD as a beneficial drug. Furgiuele et al. [47] indicate a need for *ex vivo*/*in vitro* studies in human immune cells to better analyze the targets acted upon by CBD in MS. Filippini et al. [74] have shown the benefits and safety of cannabis-based medicines for people with MS. These authors also indicate that the FDA has not approved any commercial application for CBD-based medicine for MS yet. Additionally, the FDA has been recently asked to place cannabis-based therapy for progressive MS on the fast track.

On the other hand, in 2014 the EMA approved the use of nabiximols for the management of moderate to severe spasticity in adults with MS. These patients have shown significant clinical improvement in the initial period with CBD-based therapy [74]. Moreover, it is worth underlining that in 2018 the Australian Government Department of Health recommended using cannabis-based medicines in the therapy of MS for adults who were observed to not obtain any markable improvements when treated using other antispasticity drugs [75].

### 5.1. The Role of CBD in the Treatment of Sympthoms: Spasticity and Pain

**Figure 4:**
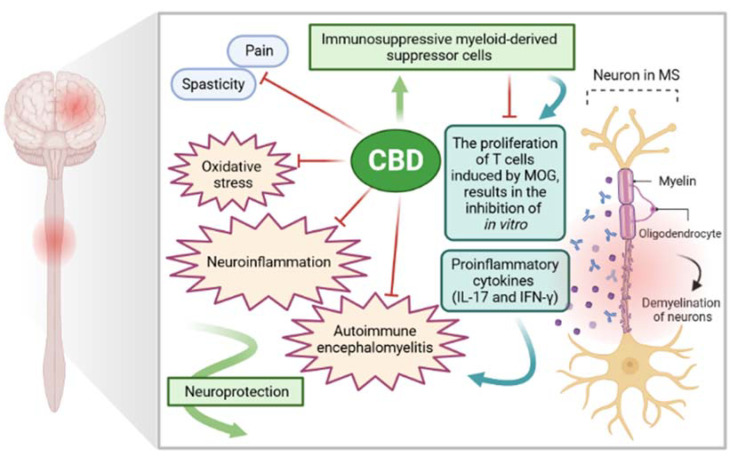
The role of CBD in the pathogenic mechanism of multiple sclerosis [own drawing]. Legends: CBD: Cannabidiol; MOG: Myelin oligodendrocyte glycoprotein; IL-17: Interleukin-17; IFN-γ: Interferon-gamma.

Several studies have been carried out to clarify the mechanisms of action through which CBD may beneficially influence MS therapy. Currently, it has not been proven that any known receptor site can be connected with CBD. Moreover, the molecular pharmacology of CBD has not been well defined yet [76]. Due to the high risk of side effects, the efficacy of treatments for MS is very limited. In the framework of different preclinical and clinical studies, cannabinoids, especially CBD, have shown immunomodulating properties. Hence, this nonpsychotropic chemical compound of *Cannabis* is commonly considered a promising anti-inflammatory and immunosuppressive agent. CBD seems to exhibit a better tolerability profile at high doses. To date, cannabis-based medicines have a great interest among MS patients to treat spasticity and pain [47]. A potential CBD-receptor-mediated signaling pathway is still unknown, but it has been confirmed that many of the CBD actions are related to both central and peripheral actions [76]. Although numerous CBD effects are related to both central and peripheral actions, the current research is focused on the careful identification of its mechanism. As described by Elliott et al. [77], CBD-based medicines for MS treatment were approved in some countries; nonetheless, the exact mechanism of action causing a reduction of neuroinflammation has not been described. To assay the anti-inflammatory CBD, Elliott et al. [77] have used a murine model of MS as an experimental autoimmune encephalomyelitis (EAE). The obtained results proved that CBD effectively attenuated the symptoms of EAE. Additionally, a significant reduction in clinical scores of paralysis decreased T cell infiltration in the CNS, and reduced levels of IL-17 and IFN-γ were observed [77,78] (Figure 4). CBD increased myeloid-derived suppressor cells (MDSCs) in EAE mice. Moreover, the high impact of MDSCs in CBD-mediated attenuation of EAE led to the inhibition of myelin oligodendrocyte glycoprotein (MOG)-induced proliferation of T cells *in vitro*. Thereby, the studies confirmed that by inducing immunosuppressive MDSCs, CBD-based treatment may minimize EAR [47,77]. Besides the results obtained in rodent models of EAE that showed efficient action of CBD, more tests and clinical trials are needed to identify the action of CBD in human immune cells [47]. To sum up, CBD-enriched therapy for MS caused the reduction of relapses in EAE. Apart from a general decrease in clinical aspects, reductions in the onset of symptoms and the rate of disease progression were also noticed [78]. In some cases, improved CNS histology was also observed [47]. Mecha et al. [71] described the beneficial impact of CBD on animal models of MS. These authors also focused on the perspectives for human treatment. In turn, Yadav et al. [79] confirmed the high effectiveness of pharmacological treatments with *Cannabis* and its derivatives to treat spasticity and pain among patients with MS.

If conventional antispastic therapy is not efficient enough, then nabiximols, commercially known as Sativex (containing CBD and THC) were mainly recommended for the treatment of pain and spasticity in MS [72], but can also be applied in inflammatory disease therapy with demyelination and neuronal injury in the brain and spinal cord. For the treatment of spasticity, Sativex was approved for the first time by the UK in 2010; however, nowadays it is recommended in more than 25 countries [47]. Interestingly, recent reports showed that the use of *Cannabis* is reported by 20–60% of patients with MS [47]. Furthermore, almost all of them would consider using it if it was legally approved [80,81].

### 5.2. Antioxidant Properties of CBD

Thanks to a polyphenolic structure, CBD belongs to the group of the most potent antioxidants. Therefore, CBD in Sativex may intensify the beneficial effects of ∆^9^-THC by reducing its psychoactive effect simultaneously. As described by Jones and Vlachou [76], the ability of CBD to inhibit the functional consequences of CB1 activation in the brain may be caused by the indirect enhancement of adenosine A1 receptors’ activity through ENT inhibition. This process can clarify the lower risk of developing psychotic symptoms in patients who use preparations based on high ∆^9^-THC and CBD ratios in comparison with patients who used a lower ratio of these cannabis-derived ingredients. When patients can tolerate higher amounts of ∆^9^-THC, Sativex attenuates spasticity and pain in MS more efficiently than a pure THC dose. It has been proven that through the enhancement of glycine signaling and the inhibition of endocannabinoid degradation, CBD can refill the antispastic effects of ∆^9^-THC [76].

## 6. Anticancer Activity of Cannabidiol (CBD)

### 6.1. Mechanisms of Action of CBD in Cancer

CBD was shown to have effects on many types of cancer. Some neural system cancers are presented in Table 2. CBD inhibits cancer cell growth and migration and affects the disruption of F-actin integrity [82,83,84,85,86,87]. Moreover, CBD inhibits migration and invasion of endothelial cells and angiogenesis. Additionally, it downregulates proangiogenic factors [88]. The mechanism of action of CBD against cancer cells may be based on several pathways.

**Figure 5:**
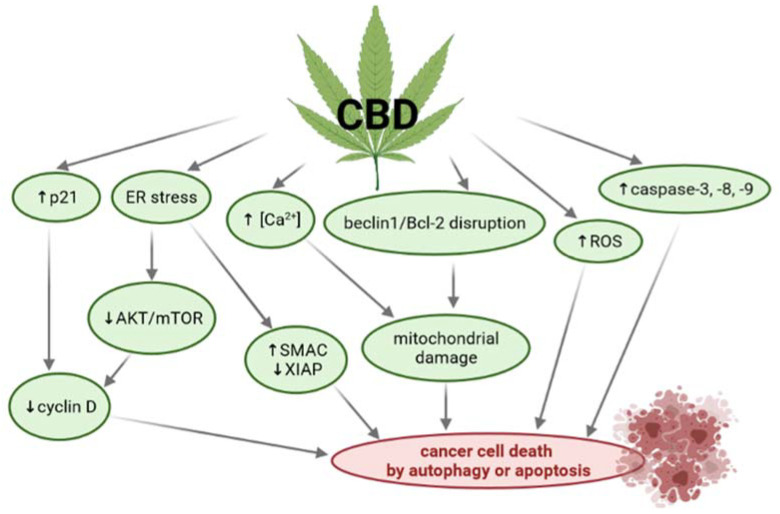
Pathways of anticancer activity of cannabidiol (CBD). Legends: AKT—Protein kinase B; Bcl-2—B-cell lymphoma 2; ER—endoplasmic reticulum; mTOR—the mechanistic target of rapamycin; p21—cyclin-dependent kinase inhibitor 1; ROS—reactive oxygen species; SMAC—second mitochondrial-derived activator of caspase; XIAP—X-linked inhibitor of apoptosis protein [own drawing].

CBD can promote CDKN1A (p21) expression in cells. Increased p21 protein expression evokes cell cycle arrest via cyclin D inhibition and the stimulation of GADD45A/B expression [83]. CBD can also induce endoplasmic reticulum (ER) stress [87,89], inhibiting AKT and mTOR signaling and subsequently decreasing the levels of phosphorylated mTOR and cyclin D1. CBD can inhibit the interaction between beclin-1 and Bcl-2, leading to reduction of mitochondrial membrane potential, mitochondrial Ca^2+^ overload, the release of cytochrome c to the cytosol, and activation of the intrinsic apoptotic pathway [89]. CBD also increases the generation of reactive oxygen species (ROS) [90,91,92]. Finally, during CBD-treatment, cancer cells can be destroyed by the induction of apoptosis and/or autophagy (Figure 5) [83,84,89,93].

### 6.2. Anticancer Effect of CBD

In the *in vitro* studies, CBD induced the decrease in glioma stemlike cell viability. The half-maximal inhibitory concentration (IC_50_) was between 14.6 μM and 19.4 μM at 24 h post-treatment. CBD in the lowest effective dose (10 μM) reduced AKT activity in glioma stemlike cells, leading to autophagy [93]. In U87 human glioma cells, the CBD treatment for 6 h resulted in a concentration-dependent migration inhibition. The IC_50_ was 5.05 ± 1.1 μM. Simultaneously, in concentrations 0.01–9 μM of CBD, no effect on cell viability was observed [85]. In other studies, CBD induced apoptosis in the U87 and U373 human glioma cell lines. In the 3-(4,5-Dimethylthiazol-2-yl)-2,5-Diphenyltetrazolium Bromide (MTT) test, CBD inhibited the mitochondrial oxidative metabolism in concentrations from 5 μM to 40 μM. The IC_50_ values were 26.2 ± 2.8 μM for U87 and 24.1 ± 2.16 μM for U373 cells [94]. In athymic nude mice with inoculated U87 cells, CBD-treated animals at day 18 had about 70% smaller and at day 23 about 50% smaller tumors than in control mice [94]. Additionally, CBD induced an increase in calcium influx in U87MG cells, with an EC_50_ value of 22.2 µM. In the U87MG glioma cell line and MZC primary glioblastoma cells, CBD affected the viability of both cells at doses > 25 µM, inducing apoptosis [95]. CBD in concentrations of 0.5–12 µM caused also decrease in U87-MG cell invasion from 10% to 90% [96]. Single studies showed an excellent therapeutic interaction of cannabidiol and γ-irradiation or chemoradiation [99,100].

In acute lymphoblastic leukemia of T lineage (T-ALL), CBD at 10 μM inhibited proliferation and at 30–100 μM induced cell death. CBD at lethal concentrations caused mitochondrial Ca^2+^ overload and mitochondrial pore formation. In Jurkat cells, one of the leukemic cell lines, 10 µM concentration of CBD induced autophagy, and 30 μM CBD caused mitochondrial damage and induced cytochrome C release from mitochondria. CBD also increased caspase-3 and -9 activity, which confirms the impact on intrinsic apoptosis [90]. Another study showed that CBD was highly cytotoxic, with an IC_50_ of 12.1 µM. CBD also triggered an increase in ROS production, when compared to untreated Jurkat cells [90,91].

CBD at concentrations of 5–50 μg/mL effectively reduced the viability of neuroblastoma cell lines (SK-N-SH25, IMR-3226, NUB-627, and LAN-1) in a dose- and time-dependent manner. Moreover, CBD led to cell arrest in the G1 phase and affected cell morphology. The cells became rounded and swollen, which confirmed that CBD induces apoptosis. In SK-N-SH cells xenograft mice treatment with 20 mg/kg of CBD suppressed tumor growth [84].

CBD induces apoptosis also in SH SY5Y and IMR-32 neuroblastoma cell lines. This activity was observed in concentrations of 5–10 µM. Apoptosis was related to the induction of caspase-2 and -3 and inhibition of mitochondrial respiration. Moreover, CBD significantly reduced migration and invasion of neuroblastoma cells [97].

The next research studied three medulloblastoma cell lines (D283, D425, and PER547) and two ependymoma cell lines (IC-1425EPN and DKFZ-EP1NS). CBD had cytotoxic activity in all cancer cell lines with EC_50_ of 3.2–4.1 µM for medulloblastomas and 7.5–10.1 µM for ependymomas. The authors observed that CBD increases ROS production. Finally, cannabidiol leads to induction of autophagy and apoptosis by MAPK and AKT/mTOR signaling [98].

Thus far, CBD is not accepted by European Medicines Agency (EMA) or U.S. Food and Drug Administration (FDA) for cancer treatment. The FDA only approved Marinol and Syndros for therapeutic uses in nausea associated with cancer chemotherapy [101].

## 7. Conclusions

In this review was presented the potential role of CBD in the treatment of some neurological diseases. The strengths of this work are that it is multidisciplinarity and presents the newest data about CBD. The limitation is the relatively small number of references, which is related to the fact that CBD has only gained popularity in recent years.

Evidence-based knowledge on the mechanisms of action of CBD in selected neurological and neoplastic diseases, including cancers of the nervous system, was provided. Insightful analysis of the results from several pharmacological studies demonstrated that CBD can be considered a promising bioactive substance from plant sources for the effective treatment of Alzheimer’s disease, epilepsy, multiple sclerosis, and neurological cancers. Recent scientific reports draw further directions for research on CBD mechanisms of action, taking into account different targets. Since some of the described mechanisms of action are still unclear, such as the antiapoptotic effects of CBD during cognitive decline or increasing the expression of autophagy-related to proteins by CBD, further studies are still in demand to draw more consistent conclusions. Thus, future studies should assess the relationship between the mechanisms of action of CBD and its therapeutic effect in clinical trials.

## Figures and Tables

**Table 1:** Summary of the mechanism of action in Alzheimer’s disease.

**The Effect of CBD on Neuroinflammatory Processes**
→ Inhibition of NF-kB (revealed by downregulation of p50 and p65); → Selective activation of PPAR-γ; → Diminishing the expression of IL-1β and iNOS; the release of NO, IL-1β, TNF-α; → Decreasing the NO generation; → Inhibiting mRNA expression of GFAP; → Increasing activity of pathways for Aβ clearance by upregulating the autophagy in the hippocampus; → Increasing the expression of autophagy-related proteins (Beclin1 and LC3); → Inhibiting the increase in the intracellular calcium induced by ATP in cultured microglia; → Diminishing the expression of proinflammatory miRNAs associated with Toll-like receptor and NF-κB signaling (elevated by LPS); → Inhibition of phosphorylated form of p38 MAP kinase; → Activation of NFκβ transcription.
**The Effect of CBD on Acetylcholinesterase (AChE) and Cholinergic System in the Brain**
→ The ability as double inhibitors by an interaction between utilized hydrogen bonds and both catalytic triad and peripheral anionic site of AChE and BuChE; → Potency to inhibit the activity of AChE; → Increasing the level of ACh; → Normalize effect in the expression of ChAT; → Normalize effect in the binding density of muscarinic M1/M4 receptor.
**Antiapoptotic Effects of CBD during Cognitive Decline**
→ Decreasing the protein level of Caspase 9, Caspase 3, cleaved PARP-1; → Inhibiting APAF1.
**The Effect of CBD on a Beta-Amyloid Synthesis**
→ Decreasing the fragments (C83 and C99) of APP; → Reducing the expression of Aβ peptide; → Activation of PPAR-γ.
**The Effect of CBD on Hyperphosphorylated Forms of Tau Protein**
→ Diminishing the expression of tau protein and decreasing the hyperphosphorylation; → Reducing of p-GSK3-β (tau protein kinase).

Legends: ACh: Acetylcholine; AChE: Acetylcholinesterase; APAF-1: Apoptotic protease activating factor 1; APP: Amyloid-beta precursor protein; BuChE: Butyrylcholinesterase; ChAT: Choline acetyltransferase; GFAB: Glial fibrillary acidic protein—marker of activated astrocytes; IL-1β: interleukin-1β, proproinflammatory cytokin; iNOS: inducible nitric oxide synthase (iNOS); NFκβ: nuclear factor kappa-light-chain-enhancer of activated B cells; PARP-1: poly (ADP-ribose) polymerase-1; p-GSK3-β: phosphorylated glycogen synthase kinase 3-β; PPAR-γ: peroxisome proliferator-activated receptor–γ.

**Table 2:** The activity of cannabidiol (CBD) against selected types of neural system cancers.

Biological Target: Cancer Cells	Dose	Activity	References
Glioma stem-like cell lines	10–25 μM	*in vitro*: Decrease in cell viability, induction of autophagy	[93]
Glioma cell line U87	0.01–9 μM	*in vitro*: Inhibition of migration, no effect on cell viability	[85]
Glioma cell line U87 and U373	20–40 μM	*in vitro*: Induction of apoptosis	[94]
Glioma cell line U87 and primary glioblastoma cells MZC	>25 µM	*in vitro*: Increase in calcium influx, reduction of viability, induction of apoptosis	[95]
Glioma cell line U87-MG	0.5–12 µM	*in vitro*: Decrease in cell invasion	[96]
Neuroblastoma cell lines SK-N-SH25, IMR-3226, NUB-627 and LAN-1	5–50 μg/mL	*in vitro*: Reduction of cell viability, induction of apoptosis	[84]
SK-N-SH cells xenograft mouse model	20 mg/kg	*in vivo*: Suppression of tumor growth	[84]
Neuroblastoma celllines SH SY5Y and IMR-32	5–10 µM	*in vitro*: Induction of apoptosis, reduction of cell migration and invasion, inhibition of mitochondrial respiration	[97]
Medulloblastoma cell lines D283, D425, and PER547	EC_50_ 3.2–4.1 µM	*in vitro*: Elevation of ROS production, induction of apoptosis and autophagy	[98]
Ependymoma cell lines IC-1425EPN and DKFZ-EP1NS	EC_50_ 7.5–10.1 µM	*in vitro*: Elevation of ROS production, induction of apoptosis and autophagy	[98]

EC_50_—Half maximal effective concentration.

## Data Availability

Not applicable.
